# P-776. Value Of Neutrophil/Lymphocyte Ratio In Predicting Relapse In Multifocal Tuberculosis

**DOI:** 10.1093/ofid/ofae631.970

**Published:** 2025-01-29

**Authors:** Fatma Hammami, Khaoula Rekik, Fatma Smaoui, Amal Chakroun, Chakib Marrakchi, Makram Koubaa, Mounir Ben Jemaa

**Affiliations:** Infectious Diseases Department, Hedi Chaker University Hospital, University of Sfax, Tunisia, Sfax, Sfax, Tunisia; Infectious Diseases Department, Hedi Chaker University Hospital, University of Sfax, Tunisia, Sfax, Sfax, Tunisia; Infectious Diseases Department, Hedi Chaker University Hospital, University of Sfax, Tunisia, Sfax, Sfax, Tunisia; Infectious Diseases Department, Hedi Chaker University Hospital, University of Sfax, Tunisia, Sfax, Sfax, Tunisia; Infectious Diseases Department, Hedi Chaker University Hospital, University of Sfax, Tunisia, Sfax, Sfax, Tunisia; Infectious Diseases Department, Hedi Chaker University Hospital, University of Sfax, Tunisia, Sfax, Sfax, Tunisia; Infectious Diseases Department, Hedi Chaker University Hospital, University of Sfax, Tunisia, Sfax, Sfax, Tunisia

## Abstract

**Background:**

Multifocal tuberculosis remains a life-threatening disease. The involvement of two non-contiguous extrapulmonary sites makes the prognosis difficult to predict. Neutrophil-lymphocyte ratio (NLR) is a useful biomarker for predicting outcome in various clinical setting. We aimed to study the value of NLR in predicting relapse in multifocal tuberculosis.

**Methods:**

We conducted a retrospective study including all patients hospitalized in the infectious disease department for multifocal tuberculosis between 1998 and 2022. The NLR was defined as the number of neutrophils divided by the number of lymphocytes in whole blood. Receiver operating characteristic curves and areas under the curves (AUC) were calculated to evaluate the predictor value of NLR.

**Results:**

We encountered 94 cases, among whom 64 were females (68.1%). Relapse was noted in 9 cases (9.6%). The NLR was significantly higher among relapsing multifocal tuberculosis cases (4.1[2.5-8.5] vs 2.4[1.6-3.7] ; p=0.031). The optimal cut-off of NLR for predicting relapse among multifocal tuberculosis cases was 3.7, with a sensitivity of 62.5% and a speciﬁcity of 75.4%. The AUC was 0.737 and the 95% conﬁdence interval ranged from 0.575 to 0.899.

**Conclusion:**

The NLR was a useful biomarker in predicting relapse among multifocal tuberculosis cases. Such patients require a close follow-up in order to promptly diagnose relapse.

**Disclosures:**

**All Authors**: No reported disclosures

